# Tuning and disrupting the brain—modulating the McGurk illusion with electrical stimulation

**DOI:** 10.3389/fnhum.2014.00533

**Published:** 2014-08-04

**Authors:** Lucas Murrins Marques, Olivia Morgan Lapenta, Lotfi B. Merabet, Nadia Bolognini, Paulo Sérgio Boggio

**Affiliations:** ^1^Social and Cognitive Neuroscience Laboratory and Developmental Disorders Program, Center for Health and Biological Sciences, Mackenzie Presbyterian UniversitySao Paulo, Brazil; ^2^Laboratory for Visual Neuroplasticity, Department of Ophthalmology, Massachusetts Eye and Ear Infirmary, Harvard Medical SchoolBoston, MA, USA; ^3^Department of Psychology, University of Milano-Bicocca, and IRCCS Istituto Auxologico ItalianoMilano, Italy

**Keywords:** McGurk illusion, superior temporal, parietal cortex, transcranial direct current stimulation, multisensory integration, speech

## Abstract

In the so-called McGurk illusion, when the synchronized presentation of the visual stimulus /ga/ is paired with the auditory stimulus /ba/, people in general hear it as /da/. Multisensory integration processing underlying this illusion seems to occur within the Superior Temporal Sulcus (STS). Herein, we present evidence demonstrating that bilateral cathodal transcranial direct current stimulation (tDCS) of this area can decrease the McGurk illusion-type responses. Additionally, we show that the manipulation of this audio-visual integrated output occurs irrespective of the number of eye-fixations on the mouth of the speaker. Bilateral anodal tDCS of the Parietal Cortex also modulates the illusion, but in the opposite manner, inducing more illusion-type responses. This is the first demonstration of using non-invasive brain stimulation to modulate multisensory speech perception in an illusory context (i.e., both increasing and decreasing illusion-type responses to a verbal audio-visual integration task). These findings provide clear evidence that both the superior temporal and parietal areas contribute to multisensory integration processing related to speech perception. Specifically, STS seems fundamental for the temporal synchronization and integration of auditory and visual inputs. For its part, posterior parietal cortex (PPC) may adjust the arrival of incoming audio and visual information to STS thereby enhancing their interaction in this latter area.

## Introduction

The McGurk effect is a crossmodal illusion which represents a by-product of multisensory integration process between auditory and visual stimuli (McGurk and MacDonald, 1976). Typically, when presented together, the perception of different stimuli category, such as the auditory bilabial syllable /ba/ and visual velar syllable /ga/, generate the perception of the integrated syllable /da/, while the opposite presentation (auditory /ga/ and visual /ba/), generates the perception of /bga/.

Numerous neuroimaging studies have identified putative cortical areas that are active during the McGurk illusion including primary and secondary auditory cortices (Pugh et al., 1996; Calvert et al., 1998; Pekkola et al., 2006), occipital cortex (Sams et al., 1991; Kushnerenko et al., 2008), prefrontal cortex (Bushara et al., 2001), superior temporal sulcus (STS; Calvert et al., 1998; Sekiyama et al., 2003; Beauchamp et al., 2010) and motor cortex (Skipper et al., 2007). Recent evidence also suggests that bilateral parietal and inferior frontal areas may also participate in the illusion, by accommodating audiovisual integrative learning processes (Kilian-Hütten et al., 2011). However, it is the STS that has been considered as the key site, playing a central role for the integration of speech and non-speech auditory and visual stimuli (Calvert et al., 1998; Saint-Amour et al., 2007; Beauchamp et al., 2010).

Although functional magnetic resonance imaging (fMRI) studies provide important evidence of brain regions underlying the McGurk illusion, new methods of neuromodulation might provide stronger corroborative evidence of causal relationships between brain regions and sensory processing and integration. Particularly, two techniques of noninvasive brain modulation have gained interest in cognitive neuroscience namely, transcranial magnetic stimulation (TMS) and transcranial direct current stimulation (tDCS). Both these techniques have been used extensively in neurocognitive studies to uncover the mechanisms of multisensory integration given their ability to modulate the activity of brain associated with cognitive behaviors (for review see Bolognini and Maravita, 2011, 2012).

For example, TMS has been used in healthy participants to establish whether a putative heteromodal area of the brain is essential for multisensory processing. Specifically with regard to the McGurk illusion, Beauchamp et al. (2010) showed that fMRI-guided TMS delivered over the STS results in a significant reduction in the perception of the McGurk illusion.

Similarly, tDCS is now receiving growing interest for its ability to facilitate multisensory interactions and modulation of unisensory processing such as vision (Antal and Paulus, 2008) and audition (Ladeira et al., 2011). tDCS is based on the application of weak direct current stimulation (usually up to 2 mA) placed over target areas. The effects are polarity-dependent. Specifically, anodal stimulation induces an enhancement of cortical excitability, while cathodal stimulation decreases it (Nitsche and Paulus, 2000; Nitsche et al., 2008). Recent evidence on tDCS facilitation of cognitive and sensory functions are as evident as any produced by rTMS to date (Wassermann and Grafman, 2005). Thus, tDCS has evolved as a promising tool for determination of the contribution of specific cortical areas to cognitive processes, also given its relatively ease of use and safety profile compared to TMS. So far, only a few studies have used this technique in multisensory research (Bolognini and Maravita, 2011). Focusing on audiovisual sensory interactions between non-speech stimuli, anodal tDCS delivered to posterior parietal cortex (PPC), facilitates crossmodal spatial orienting (Bolognini et al., 2010a,b), while cathodal and anodal tDCS of STS and of the occipital cortex both alter the sound-induced flash illusion (Shams et al., 2000) in an opposite manner (Bolognini et al., 2011). Furthermore, tDCS delivered over temporal cortex has been shown to modulate visuomotor speech perception (Lapenta et al., 2012).

Herein, we aim to assess whether tDCS can modulate multisensory audiovisual interactions in the speech domain, hence producing polarity-dependent effects on the McGurk illusion. The study of crossmodal illusions is of great interest in assessing the consequences of disrupting normal relationships among sensory cues. In particular, the McGurk illusion highlights how, in the speech domain, sensory-specific perceptual judgments concerning one sense (e.g., audition) can be dramatically affected by their interaction with other senses (e.g., vision). Therefore, we choose to perform two separate experiments in order to observe the effect of modulating the activity of target cortical areas using tDCS on illusionary type responses related to the McGurk illusion. In experiment 1, bilateral stimulation was delivered to the STS area. This region was targeted given that this area has been previously reported to play a key role in the generation of the illusion (Beauchamp et al., 2010). Both hemispheres are implicated in the illusion effect (Baynes et al., 1994; Neufeld et al., 2012). In experiment 2, bilateral stimulation was delivered to PPC area. We also targeted this region given that it is an important heteromodal area of sensory convergence and integration (Driver and Noesselt, 2008), and previous findings suggest that this area may play a role in the McGurk illusion (Kilian-Hütten et al., 2011) as well. Considering that both STS and PPC play a role in audio-visual speech perception, and in light of previous findings demonstrating successful modulation of multisensory processing in different tasks after stimulation of both areas, we hypothesized that both parietal and temporal stimulation would produce similar polarity-dependent effects, i.e., an increase and decrease of illusion-type responses after anodal and cathodal tDCS, respectively.

## Experimental procedures

### Ethics statement

The study was conducted according to the standards of the Declaration of Helsinki (World Medical Organization, 1996) and was approved by the ethics committee of the Mackenzie Presbyterian University and also by the National Ethics Committee (SISNEP, Brazil^1^). Accepted recommendations for the use and safety of non-invasive brain stimulation were applied (Rossi et al., 2009).

### Study design

We employed a randomized, and sham-controlled design to investigate the effects of a single-session of tDCS targeting the STS or PPC on a speech integration task underlined by multisensory integration process in normal healthy volunteers.

### Experiment 1

#### Participants

Twenty-four healthy participants (12 men, all right-handed), mean age of 22 ± 4 years (mean ± Standard Deviation, S.D.) were recruited from Mackenzie Presbyterian University. All participants had normal hearing and normal or corrected-to-normal vision based on self-report. Participants were regarded as suitable to participate in this study if they fulfilled the following inclusion and exclusion criteria: (1) aged between 18 and 35 years; (2) no clinically significant or unstable medical, or neuropsychiatric disorder; (3) no history of substance abuse or dependence; (4) no use of central nervous system-effective medication; (5) no history of brain surgery, tumor, or intracranial metal implantation; and (6) native Portuguese speakers. All participants provided written informed consent prior to the experiment.

#### McGurk test

Using a digital camera (*Sony Handycam DCR-HC52e*) we recorded the face (chin to forehead) of a woman speaking four syllables (/ba/,/ga/,/pa/,/ka/). To ensure the correct diction of each syllable, the person filmed was a licensed speech therapist. Following the procedure of Colin et al. (2005), each trial consisted of three repetitions of the same syllable which could be congruent or incongruent. For the congruent videos, each syllable was reproduced three times with the audio (A) correctly matching the video (V) (ba_A_/ba_v_/, ga_A_/ga_V_/, pa_A_/pa_V_/, ka_A_/ka_V_/). In the incongruent videos, each syllable was visually presented three times (e.g., video /ga/,/ga/,/ga/), in synchrony with a non-corresponding audio (e.g., audio /ba/,/ba/,/ba/). Therefore, the incongruent stimuli were ba_A_/ga_V_/, ga_A_/ba_V_/, pa_A_/ka_V_/, ka_A_/pa_V_/.

The test consisted of a sequence of 3-video trials (7 s), a central fixation point (1 s) and the response screen containing two options of syllable (2 s). The inter-trial interval was 1 s long. After the congruent stimuli ba_AV_, ga_AV_, pa_AV_, ka_AV_, the presented syllables were da/ba, ga/bga, ta/pa, pka/ka. After incongruent stimuli ba_A_/ga_V_/, ga_A_/ba_V_/, pa_A_/ka_V_/, ka_A_/pa_V_/ the presented syllables were da/ba, bga/ga, ta/pa, pka/ka,. Participants were instructed to verbally report which syllable was perceived. We decided to use a verbal response (instead of a keyboard response) to minimize the possibility of participants moving their eyes away from the screen, which could compromise the eye tracking data collected throughout the trial. All participant answers were entered in a digital table for subsequent analysis.

The entire task was composed by 56 trials (3-syllabes per trial): 28 trials were congruent and 28 incongruent. Training trials were performed at the beginning of each session and consisted of one trial (3-syllables each) of each of the four congruent pairs and the four incongruent pairs.

**Figure 1:**
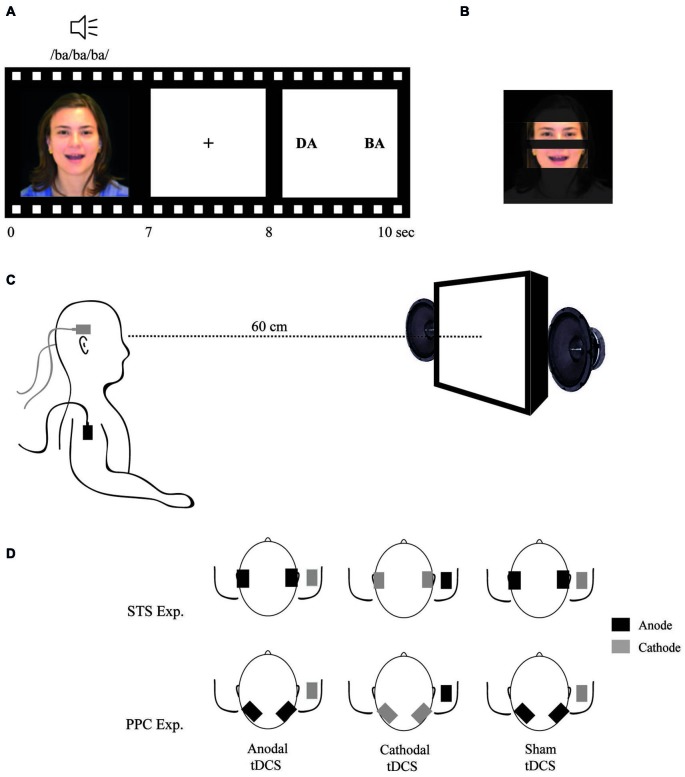
**(A)** Example of a McGurk trial; **(B)** Areas of interest (AOI) used to analyze fixation time during stimuli exposition; **(C)** Participant position; and **(D)** tDCS montages.

Stimuli were presented via a binocular eye tracking system monitor (TOBII® 1750^2^) and analyzed with the Clear View Software (version 2.5.1). Eye movements were automatically registered in real time. The eye tracking system is composed of a 17″ display (resolution of 1280 × 1024 pixels and frame rate of 60 Hz). The monitor has a built-in high-resolution camera and near infra-red light-emitting diodes (NIR-LEDs) used to track gaze direction using the pupil center corneal reflex method (Young and Sheena, 1975). For the purposes of this study, we defined the eyes and mouth regions as areas of interest (AOIs) for further fixation duration analysis (see Figure 1). Fixation duration was defined as the amount of time in which eye gaze was directed within one of the AOI’s regions.

Participants were seated at a distance of 60 cm in front of the monitor, on which a video (size of 327 × 272 pixels) was presented. The auditory stimulus intensity was set to 40 dB and auditory cues were played via two speakers positioned to the right and left of the monitor, and were visible to the participant. The picture size, audio intensity and distance from the screen were all previously determined an optimized during pilot testing prior to commencing this study. Stimuli parameters were set such that participants exhibited a detection rate of the McGurk illusion of around 70%. These parameters were then used in order to avoid potential floor and/or ceiling effects related to illusion-type responses following tDCS modulation of cortical activity.

#### Transcranial direct current stimulation (tDCS)

tDCS was delivered by two battery-driven current stimulators, each using two pairs of surface saline-soaked sponge electrodes (35 cm^2^) (Priori et al., 2008). Two electrodes (one per each device) were placed on the scalp over the temporal lobes to target the STS bilaterally and the other two were placed over the right deltoid muscle. In different sessions, all the participants received three types of stimulation:

1. Anodal tDCS: two anodal electrodes were placed over T3 and T4 (according to the 10–20 system for EEG electrode placement) and both cathodal electrodes were placed on the right deltoid muscle.
2. Cathodal tDCS: two cathodal electrodes were placed over T3 and T4 and both anodal electrodes were placed on right deltoid muscle.
3. Sham tDCS: electrodes were placed as in anodal tDCS; however, the tDCS device was turned off after 20 s of current delivery.

A constant current of 2 mA intensity was applied to the scalp at the desired areas of stimulation and to the right deltoid muscle as the reference electrode. Rubber bandages were used to hold the electrodes in place for the duration of stimulation. This bi-temporal extra-cephalic montage has been used previously and has been shown to lead to significant behavioral changes due to the bilateral stimulation (Ladeira et al., 2011; Lapenta et al., 2012).

In each session, tDCS was delivered for approximately 20 min (tDCS was started 5 min prior to and continued during the experimental task). The three tDCS sessions were randomized and counterbalanced across participants. The minimum interval between sessions was 48 h in order to avoid possible carry-over effects and to guarantee a sufficient washout period between runs. All participants tolerated the procedure well and completed the entire experiment.

### Experiment 2

#### Participants

Twenty-four healthy participants (12 men, all right-handed), mean age of 23 ± 5 years (mean ± Standard Deviation, S.D.) underwent an identical study protocol, respecting the same inclusion/exclusion criteria. In this experiment, the only difference was that now PPC was stimulated. None of the same participants performed experiment 1.

#### tDCS

Similar to experiment 1, anodal, cathodal and sham tDCS was applied bilaterally over the PPC (P3 and P4 according to the 10–20 system for EEG electrode placement), with the reference electrode positioned at the right deltoid muscle. In each session, 2 mA tDCS was delivered for 20 min (tDCS started 5 min before and continued during the experimental task). The three tDCS sessions were randomized and counterbalanced across participants. As with the first experiment, there was a minimum interval 48 h between runs.

#### Statistics

For both experiments, statistical analyses were carried out using SPSS software (version 17.7). The mean percentage of McGurk illusion-type responses were submitted to a repeated-measures analysis of variance (ANOVA), with tDCS (anodal, cathodal, sham) as main factors of interest. When appropriate, Fisher’s Least Significant Difference (LSD) *Post-Hoc* tests were performed.

Verbal responses were scored as McGurk illusion-type responses if the participant’s answer to each pair of incongruent stimuli reporting audio-visual fusion responses (McGurk and MacDonald, 1976).

In addition, an ANOVA, with tDCS as a factor, was performed for the percentage of correct responses during congruent trials as dependent variable. To investigate possible changes in eye-tracking due to tDCS, the mean duration of eye-fixation on the two AOIs (namely, eyes and mouth), was analyzed via an ANOVA with tDCS, congruence (congruent and incongruent), and AOI (mouth and eyes) as factors. When appropriate, planned pairwise comparisons were performed for the pairs Anodal vs. Cathodal, Anodal vs. Sham, and Cathodal vs. Sham.

Finally, with regard to the relation of fixation duration on the magnitude of multisensory integration, Pearson correlations were performed between illusion-type response and the fixation duration.

## Results

All the participants tolerated the stimulation well, however there were few complaints of itching sensation and mild pain (equally distributed between tDCS conditions). Regarding the STS experiment, participants reported pain in the neck (2 participants with anodal, 2 with cathodal and 1 with sham tDCS), drowsiness (1 with each condition), itching sensation under the arm electrodes (1 with anodal and 1 with cathodal). With regard to the PPC experiment, participants reported itching sensation under the arm electrodes (1 participant with anodal, 2 with cathodal and 2 with sham) and one participant reported mild headache after anodal tDCS.

Given that the experiments were planned and collected separately, the analyses were thus performed separately.

### STS experiment

#### McGurk illusion-type responses

**Figure 2:**
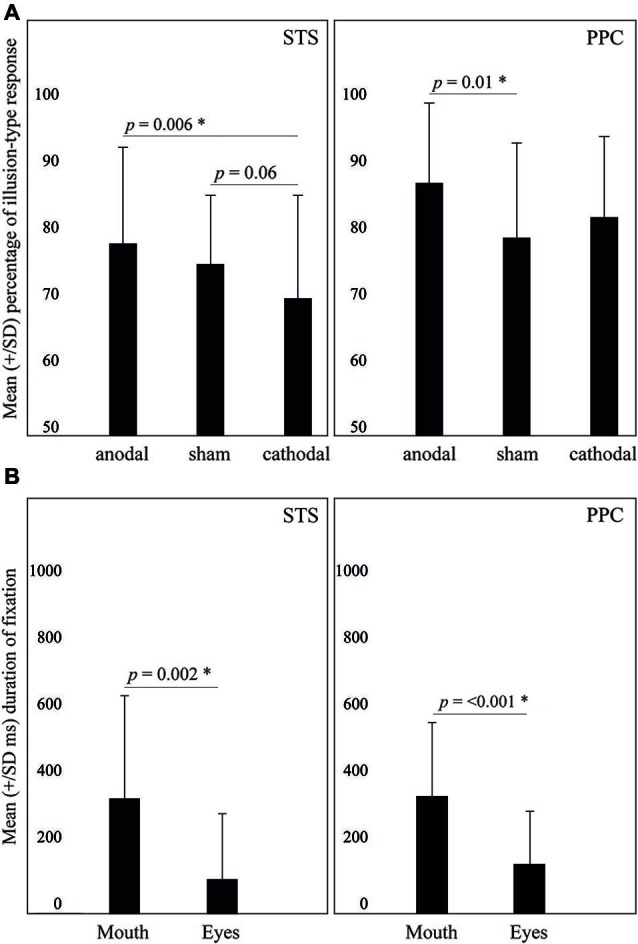
**(A)** Effects of tDCS on the McGurk effect. **(B)** Duration of fixation on the mouth or the eyes. Values are described as mean ± SD.

The ANOVA revealed a significant effect of tDCS (*F*_2,46_ = 4.34, *p* = 0.018, $\eta_{p}^{2}$ = 0.15). Fisher’s LSD *Post-hoc* showed that cathodal tDCS resulted in less illusion-type responses (70 ± 15%), as compared to anodal stimulation (78 ± 14%; *p* = 0.006). Also, there was a marginal effect when comparing cathodal to sham tDCS (75 ± 19%; *p* = 0.06) (see Figure 2). Means and standard deviation are presented at Table 1.

**Table 1:** **Percentage of correct responses during the STS experiment**.

**tDCS**	**Illusion-type stimuli**	**Congruent type-stimuli**
Anodal	78 ± 14	93 ± 4
Sham	75 ± 10	93 ± 7
Cathodal	70 ± 15	92 ± 6

*Values are described by Mean (+/−SD) of percentage*.

Finally, the ANOVA performed for the percentage of correct responses on congruent trials did not reveal significant effects of tDCS (*F*_2,46_ = 0.05, *p* = 0.95, $\eta_{p}^{2}$ = 0.001).

#### Eye-tracking

The ANOVA revealed only a significant main effect of AOI (*F*_1,23_ = 11.8, *p* = 0.002, $\eta_{p}^{2}$ = 0.3). Specifically, participants tended to fixate more the mouth area (327 ± 290 ms) as compared to the eyes (99 ± 183 ms). Other effects did not reach significance.

Additionally, Pearson correlations were performed between the percentage of illusion-type responses and the duration of fixation on the mouth for each of the tDCS montages. This analysis did not reveal significant correlations between the percentage of illusion-type responses and fixation on the mouth (Anodal: *r* = 0.3, *p* = 0.08; Cathodal: *r* = 0.1, *p* = 0.5; Sham: *r* = 0.3, *p* = 0.1).

### PPC Experiment

#### McGurk illusion-type responses

The ANOVA revealed significant effects of tDCS for incongruent trials (*F*_2,38_ = 3.41, *p* = 0.04, $\eta_{p}^{2}$ = 0.15), but not for congruent trials (*F*_2,38_ = 0.4, *p* = 0.6, $\eta_{p}^{2}$ = 0.02). Means and standard deviation are presented at Table 2. LSD *Post-hoc* showed that anodal tDCS resulted in more illusion-type responses (87 ± 12%) as compared to sham (79 ± 14%; *p* = 0.01) but not to cathodal tDCS (82 ± 12%; *p* = 0.09).

**Table 2:** **Percentage of correct responses during the PPC experiment**.

**tDCS**	**Illusion-type stimuli**	**Congruent type-stimuli**
Anodal	87 ± 12	95 ± 7
Sham	79 ± 14	96 ± 6
Cathodal	82 ± 12	97 ± 4

*Values are described by Mean (+/−SD) of percentage*.

#### Eye-tracking

With regard to eye-tracking analysis, ANOVA revealed only a significant main effect of AOI (*F*_1,19_ = 24.1, *p* < 0.001, $\eta_{p}^{2}$ = 0.5): participants fixated more the mouth (331 ± 212 ms) as compared to the eyes (139 ± 151 ms). Other effects did not reach significance.

Finally, Pearson correlations were performed between the percentage of illusion-type responses and the duration of fixation on the mouth for each tDCS montage. This analysis did not reveal significant correlations between the percentage of illusion-type responses and fixation on mouth (Anodal: *r* = −0.1, *p* = 0.6; Cathodal: *r* = −0.4, *p* = 0.8; Sham: *r* = −0.1, *p* = 0.554).

## Discussion

The present study shows that the McGurk illusion can be effectively modulated by tDCS in a polarity-specific and area-specific fashion. Specifically, anodal tDCS delivered over PPC increased the perception of the McGurk illusion. Meanwhile cathodal tDCS delivered over STS results in a marginal decrease of the illusion perception. It is notable that this modulation was specific for incongruent audio-visual speech stimuli (i.e., the illusory trial), while tDCS did not affect responses in congruent audio-visual trials (i.e., non illusory trials). Furthermore, neither anodal nor cathodal tDCS interfered with eye-tracking patterns and quantification of gaze patterns. Thus, regardless of the tDCS condition, participants fixated twice as often on the mouth area as compared to the eyes. To our knowledge, this is the first demonstration that a non-invasive brain modulation technique has been used to enhance a well-described verbal “hearing-seeing” crossmodal illusion, without changing how participants visually track the stimuli.

The tDCS effects on STS reported here are in line with previous studies. Neufeld et al. (2012) proposed that both auditory and visual differences are processed bilaterally in the STS, suggesting that this area is responsible for the joint input perception leading to the McGurk illusion (Neufeld et al., 2012). The cathodal stimulation was less effective in modulating the McGurk illusion, although a trend for a decrease of McGurk illusion-type responses is apparent. This finding points to the robustness of the McGurk illusion, which seems more resistant to the disruption brought about cathodal tDCS. In this regard, it should be also noted that, particularly when participants perform complex tasks assessing cognitive abilities, the relationships between the polarity of the tDCS, on the one hand, and the behavioral effects, on the other hand, is less straightforward, and not always the modulation of behavior by the anodal polarity (i.e., enhancement) is matched by a comparable effect in the opposite direction by the cathodal polarity (i.e., inhibition), or vice-versa (see for instance the revision by Vallar and Bolognini, 2011). Further studies are needed to confirm our findings in a larger sample. Also, we cannot exclude a possible asymmetric hemispheric contribution of STS to the McGurk illusion (e.g., Beauchamp et al., 2010; Nath and Beauchamp, 2012), as we did not assess the effects of unilateral tDCS.

Our findings are also in accordance with previous TMS findings (Beauchamp et al., 2010) pointing to STS playing a pivotal role in the perception of the illusion, but not in non-illusory (i.e., congruent) trials, as observed here. These latter authors suggest that the STS may play an important role in the temporal synchronization and integration of sensory stimuli from different modalities (Macaluso et al., 2004) given that the application of a precisely timed TMS pulse (within 200 ms after stimuli presentation) disrupted audio-visual integration. Furthermore, in line with the present findings, Bolognini et al. (2011) found that cathodal tDCS of superior temporal areas decreased the sound-induced flash illusion, while the opposite effect was found for anodal tDCS. Taken with the evidence presented here, it appears that areas along the STS integrate both speech and non-speech related stimuli, and mediate different crossmodal illusions in both, temporal processing (low-level crossmodal interactions) and speech processing (higher-level crossmodal interactions) (Noesselt et al., 2012). The fact that in the present study anodal STS stimulation did not increase the perceptual integration of incongruent stimuli may be related to a ceiling effect of the synchronization process. Concerning the lack of effect for congruent stimuli, this is also aligned with previous TMS findings of Beauchamp et al. (2010). This evidence can be explained by the fact that both unisensory modalities are receiving the same information. Therefore, even if desynchronized, perception can be directed to one of the sensory modalities without any interference.

Concerning stimulation of PPC, our data suggests that this area may play a role in the generation of the McGurk illusion (Kilian-Hütten et al., 2011). However, we found that only anodal stimulation produced modulatory effects, specifically increasing the perception of illusion-type responses. Interestingly, this is in line with the proposal of crossmodal recalibration mechanism (Fujisaki et al., 2004). The recalibration mechanism is considered necessary in order to adjust both auditory and visual information arriving from the same stimuli. This mechanism helps realign signals enabling the two modalities to be processed within specific structures responsible for their integration (Fujisaki et al., 2004; Vroomen et al., 2004). Furthermore, this process seems to occur very rapidly allowing for adjustments in different multimodal scenarios (Van der Burg et al., 2013). In a neuroimaging study by Kilian-Hütten et al. (2011), this group argues that fronto-parietal circuitry is responsible for exerting top-down influences to accommodate such a process. Therefore, the PPC might indirectly contribute to generating a more unified spatial (Macaluso et al., 2004) and temporal integration of the stimuli when the arriving auditory and visual stimuli are combined within STS. However, while being able to potentiate this audiovisual combination and thus increase illusion-type responses, PPC doesn’t seem to play a fundamental role in the integration process itself. Therefore, it seems that even when this calibration area is influenced by cathodal tDCS, frontal areas can still support this function. Another possible explanation is that the spatial asynchrony between stimuli was not sufficiently robust to pass unperceived. In turn, the STS seems crucial for the actual combination of the sensory stimuli, at least in the speech domain, and therefore its disruption by cathodal tDCS leads to an impairment of perception.

Previous findings have already shown the differential integrative aspects of STS and PPC. tDCS over PPC can modulate spatial visual-tactile interactions, but not audio-visual interactions, whereas tDCS of STS can affect temporal audio-visual interactions, but not spatial visual-tactile interactions (Bolognini et al., 2011; Convento et al., 2013). These results point to a differential involvement of parietal and temporal areas in processing specific pairings of two sensory modalities.

Since speech perception seems to be the result of audiovisual-motor integration of environmental inputs (Le Bel et al., 2009), our findings also add some important information regarding multisensory theories such as the motor theory of speech perception and the fuzzy-logical model of perception (FLMP).

The motor theory of speech perception suggests that perception of speech occurs through the “phonetic gestures”, represented in the observer’s brain as a visual motor command of the signal characterized by movements of the mouth, lips and tongue (Liberman and Mattingly, 1985). This motor activation was demonstrated by a TMS experiment that showed motor cortex activity facilitation while participants passively heard words that involved tongue movements (Fadiga et al., 2002). In turn, the FLMP proposes that auditory and visual inputs are first independently processed and then combined accordingly to the relevance of each input (Massaro et al., 1993). Thus, the integration of sensory inputs depends on the reliability of each modality which is in accordance to the Bayesian theory (Knill and Pouget, 2004).

With regards to the motor theory of speech perception, we can rule out the explanation of a sole effect of phonetic gestures. The results of cathodal tDCS over STS demonstrate that we can decrease the perception of illusory phenomena by manipulating the integration between stimuli rather than a specific aspect of the stimuli. Along these lines, a previous study from our group using the same bilateral tDCS montage over STS showed a worsening of performance from cathodal tDCS on a multisensory integration task (combining congruent and incongruent pairs of images and non-words). We found that cathodal tDCS disrupted the perception of congruent stimuli in males only (Lapenta et al., 2012). Both studies present evidence for a causal role of STS in multisensory integration. However, given that the effects were not exactly the same and we only found a marginal effect of cathodal tDCS, this suggests that the integration of different sensorial inputs might be dependent on the stimuli domain. The experiment of Lapenta et al. (2012) was composed by non-words and abstract images created to represent this invented words. Thus, the integration process might be more complex than from speech perception itself. According to Calvert (2001), the STS plays a continuous role in the integration of identity information while regions of the frontal cortex may have a more task dependent role in the perception of multiple modalities inputs. Thus, we believe that in the study of Lapenta et al. (2012), task integration recruited motor cortex and other frontal structures, while the present study relied basically on the perceptual processing of auditory and visual inputs and their integration.

Our hypothesis is also in line with Tuomainen et al. (2005) suggesting that audio-visual speech integration is unique. There is evidence that unisensory modality inputs can be perceived separately, but if perceived as speech they are integrated to form a unique perception. For example, when sine wave speech stimuli are presented without a prompt of their speech-like nature, the acoustic and visual stimuli did not form multisensory percepts. Meanwhile, when the same stimulus is presented after a speech prompt, the acoustic and visual signals combine naturally to form a coherent phonetic percept, thus yielding strong audiovisual integration. Our study participants clearly knew the speech-like nature of stimuli given that we presented the face of the speaker. Thus, we assume that participants attended to trials focused on speech perception.

Unlike TMS, tDCS does not induce action potentials (i.e., depolarizes neurons) (Nitsche et al., 2008). Rather, the enhancement and disruptive effect produced by tDCS may be the result of a modulation of spontaneous neuronal activity of the PPC and STS respectively, which represent important multisensory nodes. The possibility of improving multisensory processing by tDCS (Brunoni et al., 2012) is of great interest for its potential therapeutic application in pathological conditions characterized by impaired multisensory processing such as autism spectrum disorder and language disorders (Youse et al., 2004; Norrix et al., 2006; Dodd et al., 2008; Taylor et al., 2010; Meronen et al., 2013), given the potential behavioral benefits induced by the activation of multisensory mechanisms (Youse et al., 2004; Norrix et al., 2006; Dodd et al., 2008; Taylor et al., 2010; Bolognini et al., 2013; Meronen et al., 2013).

One limitation of our experiment is that we did not collect data from other candidate brain regions such as the inferior frontal gyri and the occipital and motor cortices as potential site of tDCS. Therefore, one could argue that our STS effects might be due to a direct interference on the primary auditory cortex rather than an effect on a specific multisensory area. This limitation is indeed plausible, particularly because tDCS electrodes are 35 cm^2^ in size and cover an extended area of the temporal lobe. Notably, Ladeira et al. (2011) found a specific effect of tDCS over the STS on a unimodal auditory temporal resolution task. Although we found a significant effect on the perception of the McGurk illusion by PPC stimulation, no significant effect was observed following both anodal STS and cathodal PPC stimulation, demonstrating that different aspects of multisensory integration depend on distinct brain regions. Therefore, modulating those regions can differently impact audiovisual integration. Our findings provide causal evidence that, although not crucial, enhancement of PPC activation can improve this type of audiovisual integration. Moreover, the nearly-significant effect of cathodal stimulation of the STS require further studies in a larger sample.

With regard to the tDCS montage, one could argue that we should focus on the left temporal cortex instead of performing a bilateral stimulation. This is also a valid argument since there is previous evidence of a left hemispheric dominance for the McGurk illusion (Nath and Beauchamp, 2012). However, in this study we choose to stimulate the temporal cortex bilaterally since there is evidence that both hemispheres play a role on perceiving the illusion (Baynes et al., 1994; Neufeld et al., 2012), and **a** previous study (Ladeira et al., 2011) has also shown that bilateral tDCS can modulate a modal auditory temporal resolution task. Moreover, Szycik et al. (2012) propose that successful audiovisual speech fusion is related to bilateral activity of the STS. Thus, in this study, we choose a bilateral montage, trying to maximize the modulatory effect of tDCS, increasing or decreasing the cortical activity bilaterally and in the same direction, thus avoiding inter-hemispheric compensatory effects. Additionally, our montage avoids the use of the reference electrode place over a cephalic area (placed over the right deltoid muscle in this study). At the same time, we have to consider that our findings did not allow us to infer whether both hemispheres were crucial to the McGurk illusion. Therefore, future studies should investigate the effects of tDCS on each hemisphere separately.

In conclusion, we showed that tDCS could enhance or diminish the perception of an audiovisual speech illusion, depending of tDCS polarity and brain region. These effects were not correlated with fixation on specific AOI showing that the possible mechanisms of tDCS on the illusion perception are not suggestive of an effect on the eye-gaze behavior, but rather on mechanisms related to multisensory integration processing.

## Author contributions

Paulo Sérgio Boggio developed the study concept. All authors contributed to the study design. Data collection was performed by Lucas Murrins Marques, Olivia Morgan Lapenta. All authors contributed to data interpretation. All authors contributed to manuscript writing and approved the final version of the manuscript for submission.

## Conflict of interest statement

The authors declare that the research was conducted in the absence of any commercial or financial relationships that could be construed as a potential conflict of interest.

## Footnotes

^1^http://portal.saude.gov.br/sisnep

^2^http://www.tobii.com

## Abbreviations

tDCS: Transcranial direct current stimulation
RGDT: Random gap detection test
A1: Primary auditory cortex
M1: Primary motor cortex
V1: Primary visual cortex
STS: Superior Temporal Sulcus.
